# Mesoporous Particle Embedded Nanofibrous Scaffolds Sustain Biological Factors for Tendon Tissue Engineering

**DOI:** 10.1021/acsmaterialsau.3c00012

**Published:** 2023-07-24

**Authors:** Chiara Rinoldi, Ewa Kijeńska-Gawrońska, Marcin Heljak, Jakub Jaroszewicz, Artur Kamiński, Ali Khademhosseini, Ali Tamayol, Wojciech Swieszkowski

**Affiliations:** † Faculty of Materials Science and Engineering, Warsaw University of Technology, Warsaw 02-507, Poland; ‡ Institute of Fundamental Technological Research, Polish Academy of Sciences, Warsaw 02-106, Poland; § Centre for Advanced Materials and Technologies CEZAMAT, Warsaw University of Technology, Warsaw 02-822, Poland; ∥ Department of Transplantology and Central Tissue Bank, Medical University of Warsaw, Warsaw 02-091, Poland; ⊥ Department of Bioengineering, University of California, Los Angeles, California 90095, United States; # California NanoSystems Institute, University of California, Los Angeles, California 90095, United States; ∇ Terasaki Institute for Biomedical Innovation, Los Angeles, California 90024, United States; ○ Department of Mechanical and Materials Engineering, University of Nebraska, Lincoln, Nebraska 68588, United States; ◆ Department of Biomedical Engineering, University of Connecticut Health Center, Farmington, Connecticut 06030, United States

**Keywords:** bead-on-string, electrospinning, wet-spinning, cell alignment, growth factors release, tendon tissue engineering

## Abstract

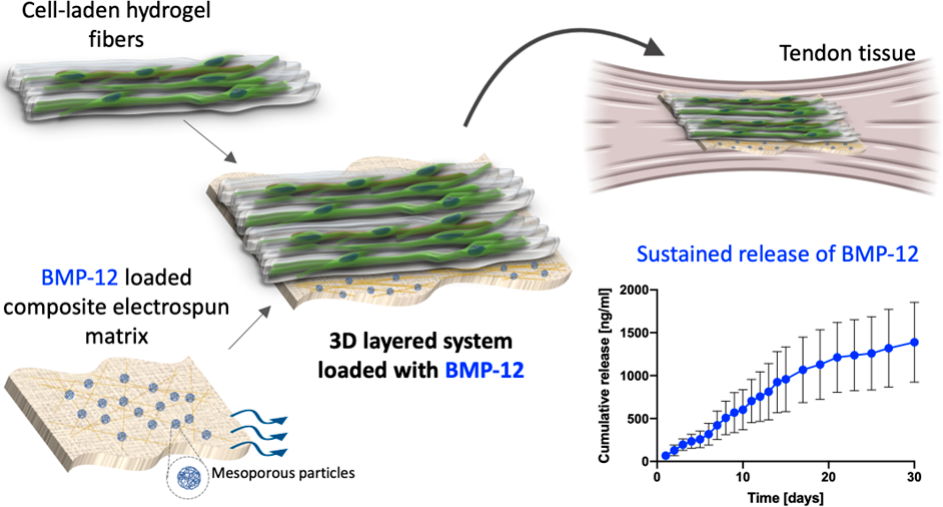

In recent years, fiber-based systems have been explored in the frame of tissue engineering due to their robustness in recapitulating the architecture and mechanical properties of native tissues. Such scaffolds offer anisotropic architecture capable of reproducing the native collagen fibers’ orientation and distribution. Moreover, fibrous constructs might provide a biomimetic environment for cell encapsulation and proliferation as well as influence their orientation and distribution. In this work, we combine two fiber fabrication techniques, such as electrospinning and wet-spinning, in order to obtain novel cell-laden 3D fibrous layered scaffolds which can simultaneously provide: (i) mechanical support; (ii) suitable microenvironment for 3D cell encapsulation; and (iii) loading and sustained release of growth factors for promoting the differentiation of human bone marrow-derived mesenchymal stem cells (hB-MSCs). The constructs are formed from wet-spun hydrogel fibers loaded with hB-MSCs deposited on a fibrous composite electrospun matrix made of polycaprolactone, polyamide 6, and mesoporous silica nanoparticles enriched with bone morphogenetic protein-12 (BMP-12). Morphological and mechanical characterizations of the structures were carried out, and the growth factor release was assessed. The biological response in terms of cell viability, alignment, differentiation, and extracellular matrix production was investigated. *Ex vivo* testing of the layered structure was performed to prove the layers’ integrity when subjected to mechanical stretching in the physiological range. The results reveal that 3D layered scaffolds can be proposed as valid candidates for tendon tissue engineering.

Tendon injuries and degenerative processes might affect the population at each age, resulting in high-cost associated procedures, which count annually about 30 million surgeries.^1^ Tissue engineering (TE) aims to overcome the disadvantages of existing treatment procedures (such as autografts, allografts, and prostheses) by designing biomimetic scaffolds and developing strategies for tissue repair and regeneration.^2−4^ The most crucial aspect of TE is the design of scaffolds that can be easily and precisely tailored in terms of structural, morphological, and mechanical properties while recapitulating the native tissue’s physiological conditions.^5−7^ In this frame, many scaffold fabrication methods, as well as synthetic and natural-based biomaterials, have been explored for tendon tissue engineering. Fiber-based systems are considered among the most promising candidates due to their ability to mimic the collagen’s fibrillar structure and architecture, as well as guide cells’ distribution, alignment, and orientation.^8−10^ Synthetic electrospun fibers have been widely utilized for tendon applications due to important advantages such as fine nanofibrous structures and interconnected pores combined with desirable mechanical properties.^11−13^ However, the lack of cell binding sites of the synthetic polymeric structures, slow degradation rate, and small pores resulted in a poor biological response, limiting their use.^14,15^ On the other hand, natural-based hydrogel fibrous constructs are considered great candidates for 3D cell encapsulation, growth, and orientation; however, they are generally insufficient to recapitulate the mechanical properties of native tendons adequately and cannot direct the organization of cells.^16−18^ For this reason, fibrous multilayered systems that combine different biomaterials and/or fabrication techniques have gained the attention of researchers.^19−22^ This approach allows combining the beneficial aspects of each compartment, obtaining ideal scaffolds which can potentially provide both mechanical support and a 3D biomimetic microenvironment.^23−25^

Mesenchymal stem cells are considered one of the best choices to be encapsulated into systems for tendon regeneration due to their potential to differentiate into tenogenic lines when treated with adequate stimuli.^26,27^ Recently, it has been demonstrated that tenogenic growth factors (GFs) play a crucial role in inducing and promoting cell differentiation toward tendons.^28−30^ In this frame, some studies have proven that a few nanograms of BMP-12 supplemented in culture media led to efficient tenogenic differentiation.^31−33^ Besides, novel strategies and approaches for avoiding the external systematic provision of growth factors during the culture/implantation time while maintaining the GF bioactivity and sustained supplement have been explored.^11,34^

In this work, we propose a cell-laden 3D layered fibrous scaffold composed of aligned wet-spun hydrogel fibers deposited on an electrospun composite matrix loaded with a tenogenic growth factor. The oriented organization of the hydrogel fibers is expected to mimic the linearity of the tendon tissue, while the electrospun fibrous component is expected to provide mechanical support and loading of bone morphogenetic protein-12 (BMP-12). The viability, distribution, maturation, and differentiation of human bone marrow mesenchymal stem cells (hBM-MSCs) encapsulated in the hydrogel fibers were investigated. *Ex vivo* mechanical stretching was applied to the 3D layered construct to evaluate the system’s response under physiological loads.

## Results and Discussion

A suitable scaffold for tendon TE should provide adequate mechanical support and direct cellular organization to mimic their axial and longitudinal directions in the native tissue. Moreover, since tenocytes are hard to harvest and culture, identifying other suitable cell sources to populate the final scaffold is extremely important.

**Figure 1:**
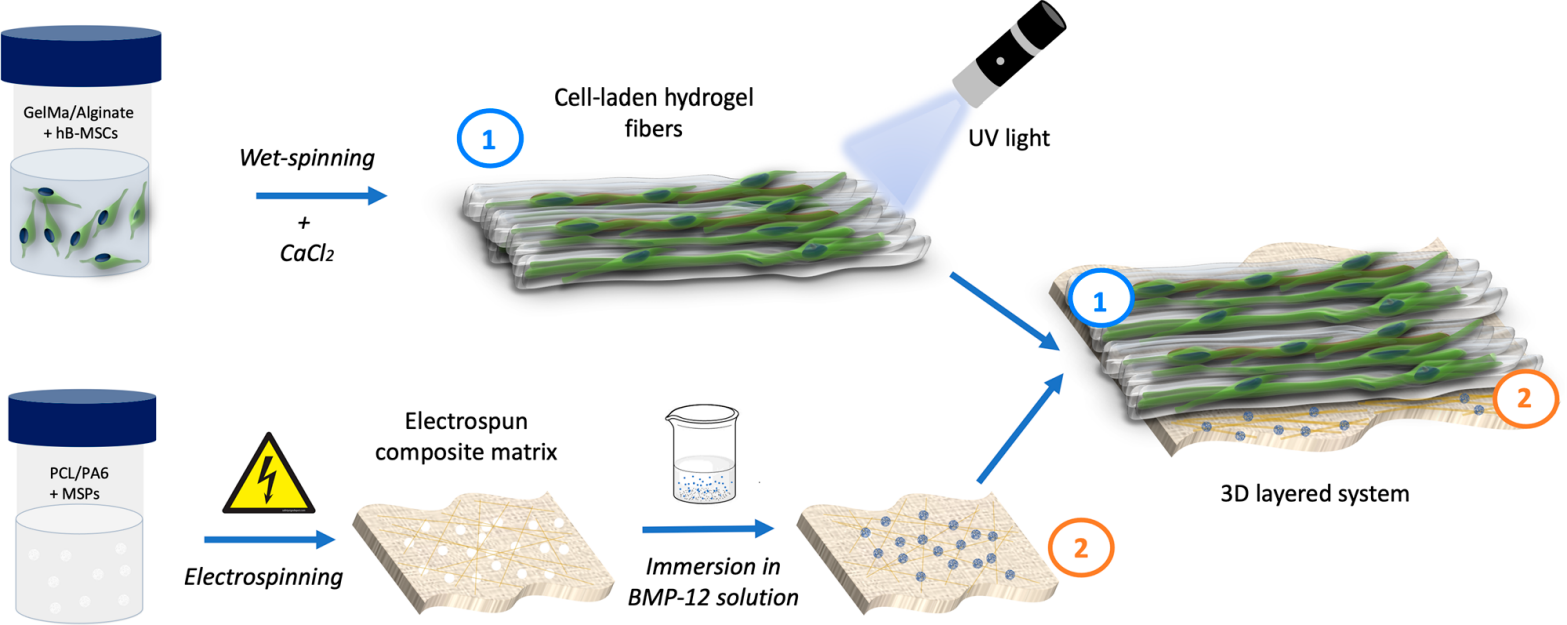
Schematic of the 3D layered scaffold fabrication. Briefly, GelMA/Alginate solution was loaded with hBM-MSCs and wet-spun in the presence of calcium chloride to obtain aligned hydrogel fibers. The fibers were then fully cross-linked by exposure to UV light and deposited onto an electrospun composite mat composed of PCL, PA6, and MSPs loaded with BMP-12 in order to form a cell-laden 3D layered fibrous system. (Numbers on the images indicate the layers: (1) hydrogel fibers layer; (2) electrospun matrix layer.)

In this work, we have combined two fiber-based fabrication techniques, namely, electrospinning, and wet-spinning, to design and produce for the first time a 3D layered scaffold formed from electrospun composite mats and cell-laden aligned hydrogel wet-spun fibers for tendon tissue engineering (Figure 1). Human bone marrow-derived mesenchymal stem cells were chosen among various cell sources to be encapsulated into the hydrogel fibers for their superior capacity to differentiate toward tendons if treated with tenogenic growth factors (*e.g.*, BMP-12).^20^

**Figure 2:**
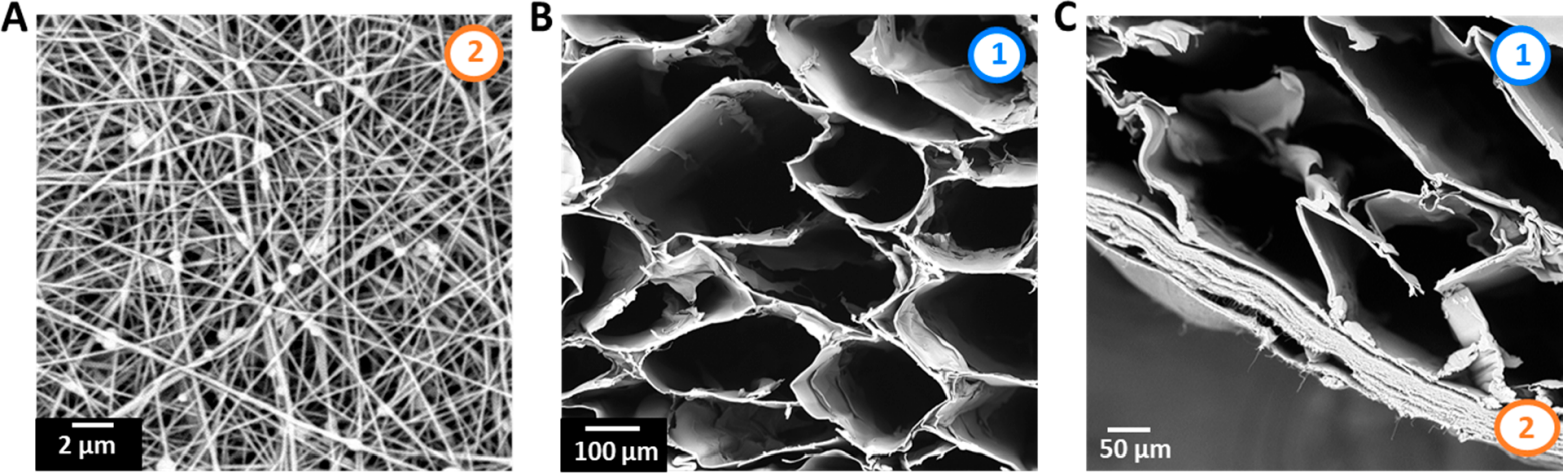
Morphological properties. SEM images: (A) electrospun composite nanofibers of PCL, PA6, and MSPs; (B) cross section of highly aligned GelMA/Alginate hydrogel fibers; (C) cross section of the layered system composed of hydrogel fibers deposited on the electrospun matrix (numbers on the images indicate the layers: (1) hydrogel fibers layer; (2) electrospun matrix layer).

The fibrous matrix was electrospun from a polycaprolactone (PCL), polyamide 6 (PA6), and mesoporous silica particles (MSPs) solution. The bigger dimension of MSPs (∼200 nm) compared to fibers’ diameters (∼180 nm) permitted the formation of homogeneous and defect-free bead-on-string architecture, with a uniform dispersion of encapsulated MSPs, as reported by Scanning Electron Microscope (SEM) images (Figure 2A).^35^ After fabrication, fibers were immersed into a BMP-12 solution to incorporate tenogenic growth factors into the system and induce the differentiation of hBM-MSCs. This simple loading method aimed to replace growth factor loading prior to electrospinning, avoiding exposure of BMP-12 to high voltage and harsh solvents during the scaffold production process, which can negatively affect its bioactivity.

On the other hand, a solution of gelatin methacryloyl (GelMA) and Alginate loaded with hBM-MSCs was wet-spun in the presence of calcium chloride. The Alginate component in the hydrogel precursor solution is essential to allow the instantaneous gelation of the bioink when in contact with calcium chloride during the fiber extrusion through a coaxial nozzle. Afterward, the obtained fibers were exposed to ultraviolet (UV) light to permit full cross-linking of the GelMA component in the hydrogel fibers. The wet-spinning setup provides a microfluidic coaxial extruder and a stepper motor which allows the obtainment of highly aligned hydrogel fibers of around 140 μm in diameter (Figure 2B), as previously described.^36^ Wet-spun hydrogel fibers were then deposited onto the electrospun mats, as shown in the SEM images of the cross-section reported in Figure 2C; finally, the hydrogel structures were secured on the nanofibrous substrate by being covered with a layer of GelMA.

**Figure 3:**
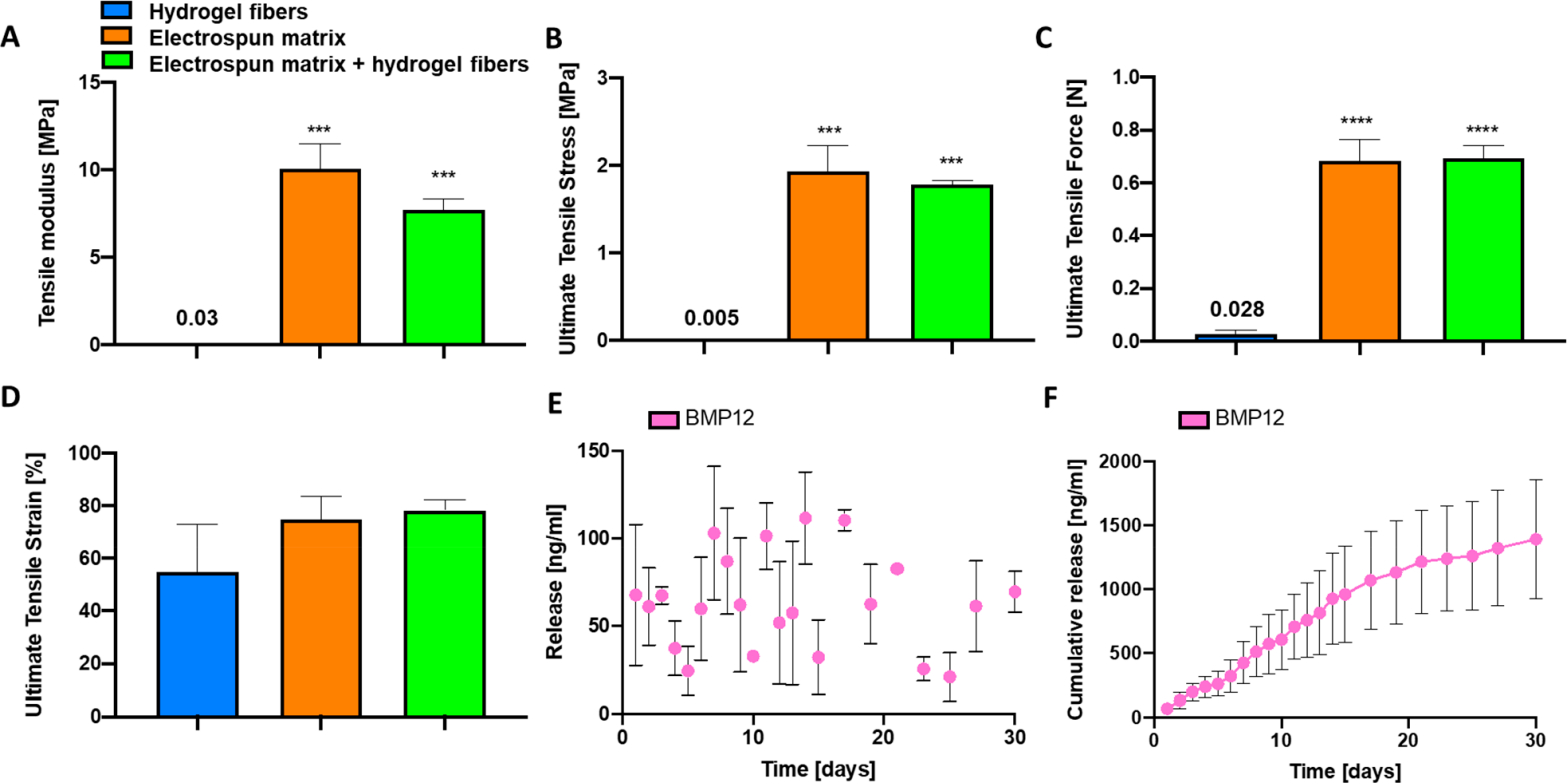
Mechanical characteristics and release profile. (A–D) Mechanical properties of hydrogel fibers, electrospun matrix, and electrospun matrix covered with hydrogel fibers: tensile modulus (A), ultimate tensile stress (B), ultimate tensile force (C), and ultimate tensile strain (D). (E,F) Release of BMP-12 from the electrospun matrix: daily release (E) and cumulative release (F). Significant differences are presented compared to the hydrogel fibers condition: *p ≤ 0.05, **p ≤ 0.01, ***p ≤ 0.001, and ****p ≤ 0.0001.

The electrospun matrix composition and fabrication parameters were optimized to provide sufficient mechanical properties to support the proper function of scaffolds postimplantation at the sites of tendon injury. Mechanical testing of the samples demonstrated that the tensile modulus and ultimate tensile stress of the composite matrix were ∼10 MPa and ∼2 MPa, respectively (Figure 3A,B). These values are on the same order of magnitude as the characteristics of the native supraspinatus tendon (tensile modulus = 40–170 MPa; ultimate tensile stress = 4.1–16.5 MPa according to the location of the selected tissue strip).^37^ On the other hand, hydrogel fibers have 1000-fold lower mechanical properties (tensile modulus = 30.7 ± 5.7 kPa; ultimate tensile stress = 4.9 ± 1.4 kPa), which appeared definitively insufficient to recapitulate the native tissue features. However, considering that hydrogel fibers are not strong enough to stand the force needed to grip the layered system in clamps, it turned out that it is not possible to perform the tensile test in that case. In such a situation, it was decided to study the effect of the presence of hydrogel fibers on the properties of the electrospun substrate. To this point, the electrospun substrate covered with hydrogel fibers was gripped as presented in Figure S1. Data show only slightly lower values of the tensile modulus and the ultimate tensile stress of the electrospun matrix covered with hydrogel fibers compared to the sole electrospun matrix. However, the observed differences are statistically nonsignificant, as also supported by comparable values of ultimate tensile force (Figure 3C). The ultimate tensile strain of the structures was also evaluated, reporting comparable values among the different conditions tested (in the range of 55–75%, Figure 3D). Results indicate no effect of hydrogel fibers on the mechanical properties of electrospun matrix, demonstrating that the electrospun component acts as mechanical support to the final scaffold.^20^ Additionally, during the mechanical test, no detachment or delamination of the hydrogel fibers on the electrospun matrix was observed.

In order to test the biological response of the scaffold, hBM-MSCs were encapsulated into the highly aligned hydrogel fibers; meanwhile, the composite electrospun matrix was loaded with BMP-12 to influence the cell fate of hBM-MSCs into tenogenic phenotype.^38^

The immersion of the composite matrix in a solution of BMP-12 for 24 h was sufficient to load the growth factors into the structure. The affinity of growth factors with the ceramic component and its intrinsic mesoporous structure most probably promoted an efficient absorption and loading of BMP-12.^39−42^ Subsequently, the matrix was incubated and the long-term release of BMP-12 was measured. The release profile, reported in Figure 3E,F revealed a sustained linear release of BMP-12 into the surrounding environment of about 60 ng/mL/day up to 30 days, which is considered sufficient to promote differentiation of MSCs into tenogenic lines, as previously reported.^20^ It is worth underlining that the proposed system favored the linear and continuous release of loaded growth factors, while several studies often reported phenomena of burst release in the first hours of incubation, exhausting the bioactive molecules in the short term.^28,43−46^ On the other hand, pristine polymeric fibrous systems suffer from low loading capacity and consequent lower, less linear, and sustained release of growth factors over time (Figure S2). Herein, thanks to the use of exposed mesoporous ceramic particles in the composite matrix, we achieved a linear and sustained release profile of growth factors in a relatively long-term.^47^ For this reason, we speculate that the system can be possibly implanted in the host body and potentially assist the regeneration of tendon tissue during the healing time, providing a continuous and sufficient amount of growth factors for the differentiation of the inner and surrounding cells.

Thus, the biological response of hBM-MSCs encapsulated within the hydrogel fibers, 3D layered scaffold, and 3D layered scaffold enriched with BMP-12 was assessed, evaluating cell viability, orientation, and differentiation as well as collagen production. To prove that the viability of hBM-MSCs was maintained after encapsulation in the proposed layered system, a Live/Dead kit was assayed 24 h after the fabrication process. Fluorescence images showed live cells in green color and nonviable cells stained in red (Figure S3A), highlighting that most of the cells were alive (>88%, Figure S3B). Consistently, there was no significant difference among the different scaffolds. The results pointed out the preservation of cell vitality, proving the suitability of the combination of electrospinning and wet-spinning methods for fabricating constructs for tissue engineering.

**Figure 4:**
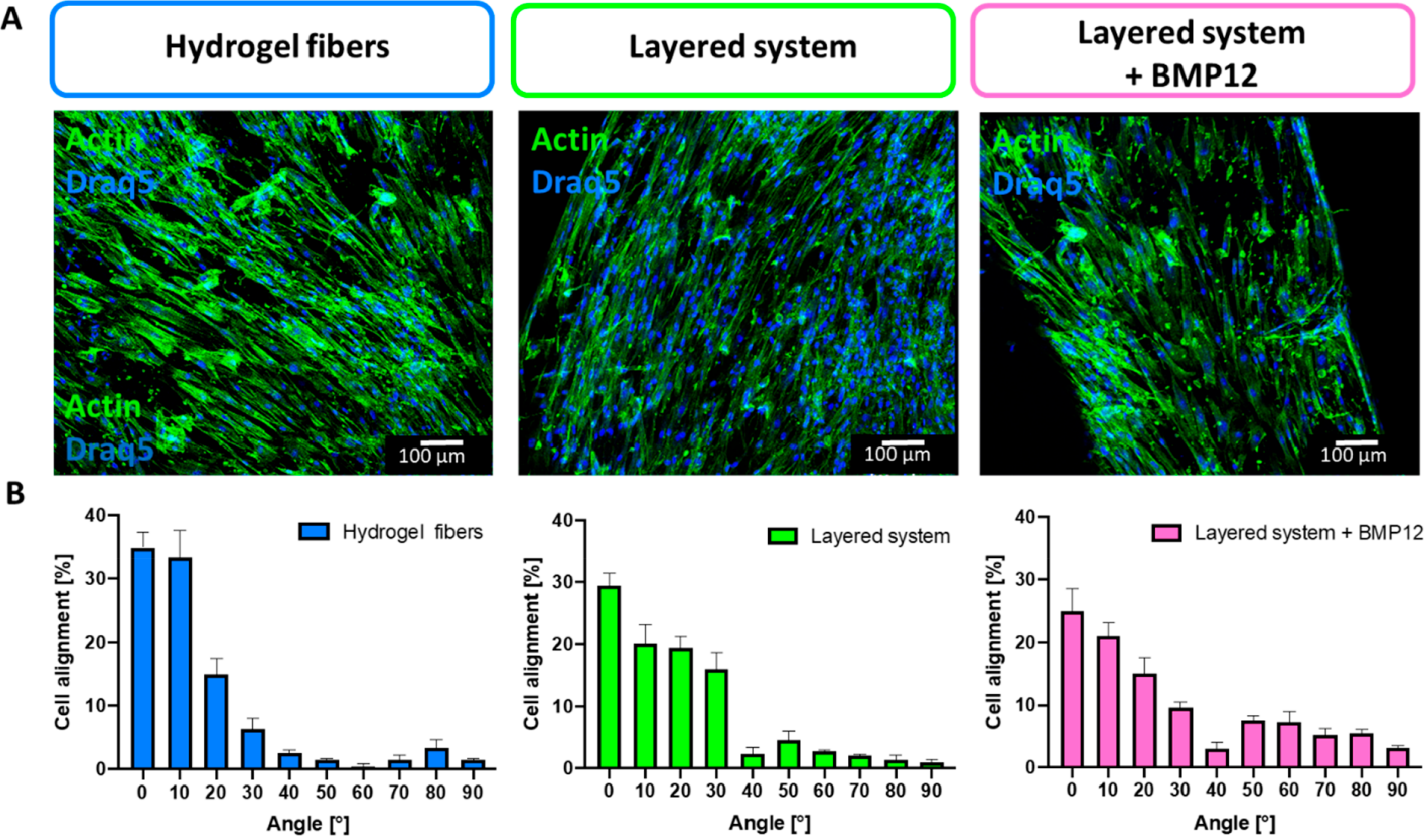
Morphology of mesenchymal stem cells encapsulated within the hydrogel fibers, 3D layered system, and 3D layered system loaded with BMP-12 after 14 days of culture. (A) Confocal images of Actin/DRAQ5 staining. (B) Quantification of cell alignment (0° is the direction of the fiber axis).

Furthermore, the highly aligned architecture of the hydrogel fibers promoted longitudinally oriented cell spreading and 3D distribution. In Figure 4A, one can observe the orientation of the cell cytoskeletons in the fiber axis direction. Considering the random distribution and orientation of hBM-MSCs into bulk hydrogels reported in a previous study,^20^ the cell alignment was probably stimulated by the orientation of the bioink polymer chains at the molecular level occurring during the extrusion process of the hydrogel fibers. hBM-MSCs loaded into fibers and cultured for 14 days formed a highly longitudinal-oriented 3D cell substrate, as previously reported.^36^ The quantification of the cell orientation angle (Figure 4B) showed that cells were aligned up to 35% in the direction of the fiber axis, mimicking the physiological tendon tissue anisotropy. Cell morphology appeared comparable in the case of hydrogel fibers deposited onto the polymeric mats or after the BMP-12 treatment.

**Figure 5:**
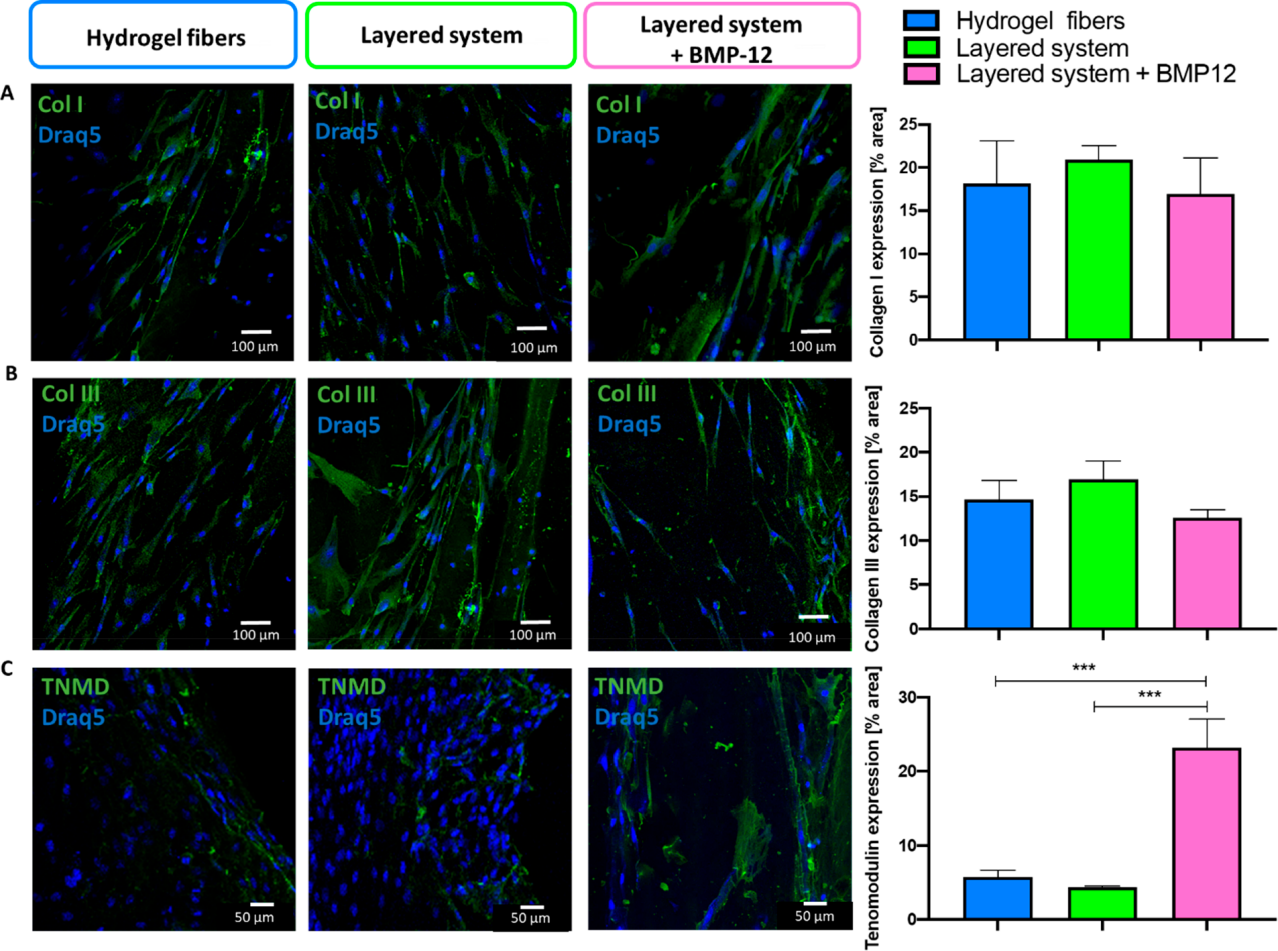
Biological performance of mesenchymal stem cells encapsulated within hydrogel fibers, 3D layered system, and 3D layered system loaded with BMP-12 after 14 days of culture. Confocal images of collagen I (A), collagen III (B), and tenomodulin (C) expressed by hBM-MSCs and the respective quantification of protein production. Significant differences are reported as *p ≤ 0.05, **p ≤ 0.01, ***p ≤ 0.001.

Additionally, the function and maturation of the hBM-MSCs loaded into the scaffolds were investigated. The specific tendon-like ECM deposition was assessed through immunocytochemistry of collagen I and III, which are known as the primary protein components of the tendon ECM.^48^ All the tested conditions reported an oriented and abundant pericellular secretion of both proteins, without a significant difference among the different conditions tested (Figure 5A,B). The collagen orientation is most probably related to the alignment of the cells, since collagen expressed by hBM-MSCs randomly distributed in a bulk hydrogel did not show preferential orientation.^20^ Thus, aligned cell-laden fibers may promote and induce the formation and deposition of organized collagen fibers, which can potentially recapitulate the native tendon ECM-oriented architecture. Finally, tenomodulin (TNMD) expression, as the late tenogenic marker playing a crucial role during tendon maturation, was investigated and quantified to explore the potential tendon differentiation of hBM-MSCs. Results showed that systems loaded with BMP-12 reported a significantly higher TNMD production level compared to untreated samples (Figure 5C). These outcomes confirmed the effective BMP-12 release from the composite electrospun component of the layered scaffold and its positive effect in promoting cell differentiation and tenogenic marker expression.^31^

**Figure 6:**
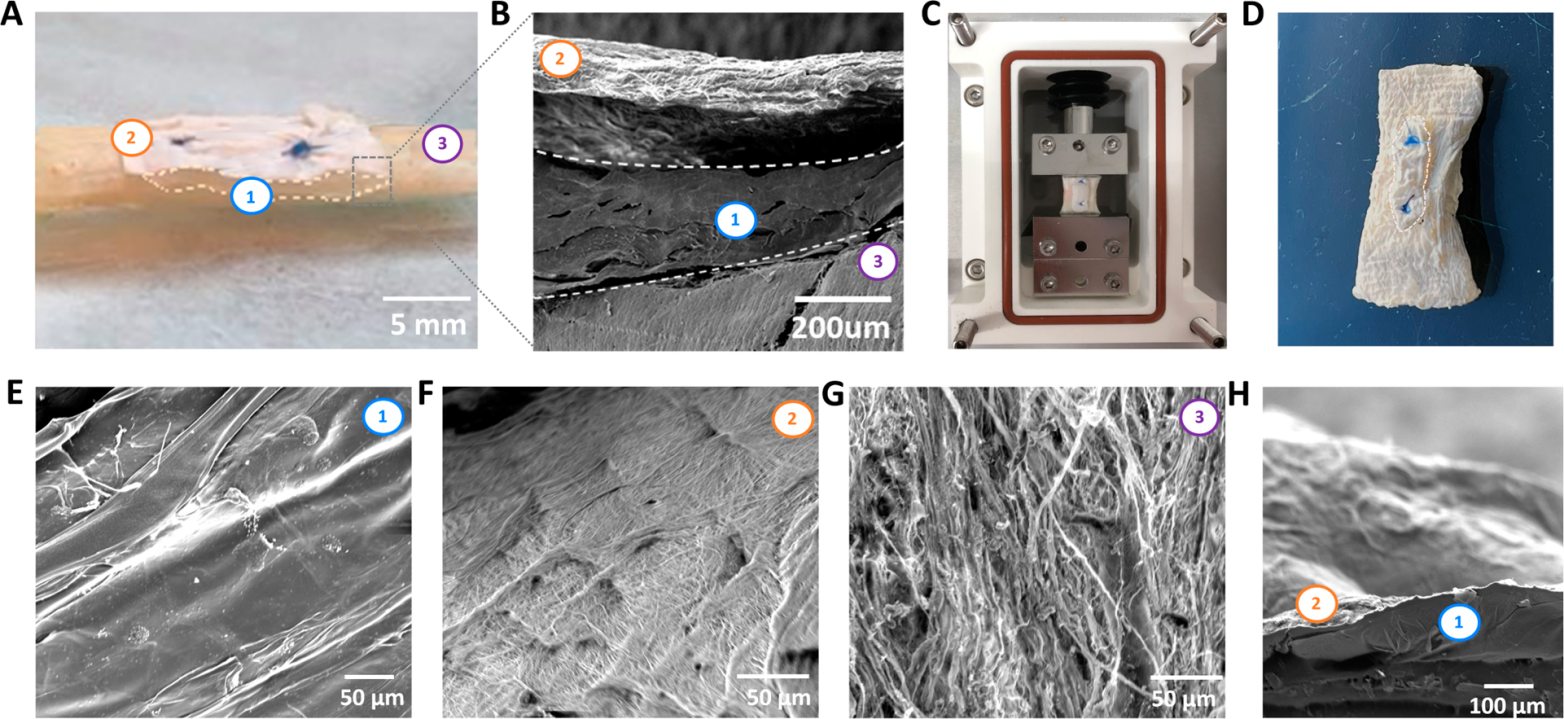
*Ex vivo* testing of the 3D layered system. (A) Suturing of the 3D layered system onto human Achilles tendon. (B) SEM image of the cross section of the construct. (C) Loading the system into a bioreactor chamber for application of cyclic tensile stretching. (D) Macroscopic photo of the dried 3D layered system sutured onto native tendon tissue after cyclic tensile stretching. (E–G) SEM images of the layers of the 3D layered system sutured on tendon tissue subjected to cyclic tensile stretching: hydrogel fibers layer (E), electrospun matrix layer (F), and tendon tissue (G), showing the maintained integrity of the layers after mechanical loading. (H) Cross section of the 3D layered system subjected to cyclic tensile stretching. (Numbers on the images indicate the layers: (1) hydrogel fibers layer; (2) electrospun matrix layer; (3) tendon tissue.)

Finally, the system was tested *ex vivo* in order to investigate its potential as a tendon substitute. In this frame, the integrity of the 3D layered system was evaluated when sutured onto human Achilles tendon and subjected to loads mimicking the physiological conditions *in vivo* during normal walking (stretching cycles between 2% and 4% strain for 5000 cycles at 1.4 Hz).^49^ The construct was surgically sutured to the native tendon from a human donor with two stitches in order to immobilize it on the tissue (Figure 6A). SEM images of the cross-section of the resulting construct showed the well-defined layered structure of the system, visualizing the electrospun mat, hydrogel fibers, and tendon tissue layers (Figure 6B). The sample was then loaded into a bioreactor chamber and placed between two grips (Figure 6C) prior to applying cyclic mechanical stretching to simulate the physiological load condition during walking. The samples were dried and analyzed after mechanical stimulation in order to investigate the influence of stretching on the integrity of the scaffold’s layers. Evident macroscopic damage or platform cracks were not observed (Figures 6D and S4). Additionally, the SEM images displayed in Figure 6E–H show the intact structure of each layer of the system after cyclic mechanical stimulation. No damage, break, or failure of the hydrogel layer (Figure 6E), the electrospun matrix layer (Figure 6F), or the tendon tissue (Figure 6G) was reported. Results are consistent and supported by the mechanical properties of the layers presented in Figure 3D, which showed the ultimate tensile strain at much higher values (>55%) than the maximum strain applied during mechanical cyclic stimulation (4%). Additionally, the cross section of the system did not show layer delamination (Figure 6H), demonstrating the maintained integrity of the layers when subjected to physiological loads *ex vivo*. Thus, the results revealed that the 3D layered system is mechanically functional as a scaffold for tendon tissue engineering and can support *ex vivo* cyclic physiological loads without failure. However, *in vivo* experiments are necessary to determine the effective abilities for tendon tissue regeneration.

## Conclusions

In this work, we produced and characterized cell-laden 3D fibrous layered systems combining for the first time an electrospun composite nanofibrous matrix and wet-spun hydrogel fibers for tendon TE. The composite mesh aimed to provide mechanical support to the construct, while the hydrogel layer was meant to create a suitable microenvironment for cell attachment and proliferation. Moreover, the electrospun composite substrate was enriched with BMP-12 to induce the tenogenic differentiation of hBM-MSCs encapsulated within the 3D layered system. Our results showed the effective linear sustained release of BMP-12 up to 30 days, and higher expression of tenogenic markers (*i.e.*, tenomodulin) was detected in the presence of BMP-12, revealing its positive effect in inducing cell differentiation toward tenogenic lines. Furthermore, hBM-MSCs cultured within oriented hydrogel fibers result in a highly aligned cell distribution, mimicking the tendon native tissue anisotropy. Finally, *ex vivo* mechanical stretching of the construct showed the well-maintained integrity of the layers under physiological loads, revealing the potential of the system for tendon tissue engineering.

Finally, it is worth mentioning that this study holds high potential for several other applications in tissue engineering. The simple GF loading method makes the proposed system highly versatile and available to be loaded with any growth factors or small bioactive molecules that can show affinity with the silica component, thus permitting the promotion of cell differentiation toward different lines and engineering of other tissues. Perspectives are related to the design of GF gradients through the construct by immersing only one part of the composite matrix into the GF solution, allowing the absorption of the bioactive molecules from one edge only. In the same manner, multiple GFs can be loaded into the scaffold in an organized manner by immersing the opposite extremity of the scaffold in solutions of two or more different GFs according to the needs of the targeted tissues. This paves the way for the fabrication of gradient scaffolds that can find their application in more complex tissue engineering applications (*e.g.*, tendon–bone and tendon–muscle interfaces).

## Experimental Section

### Materials

All the chemicals, including PCL (*M*_n_ 80000), PA6 (Nylon-6), MSPs (200 nm particle size, pore size 4 nm), acetic acid (98%), formic (99.7%) acid, methacrylic anhydride, gelatin (Type A, 300 bloom from porcine skin), 2-hydroxy-4′-(2-hydroxyethoxy)-2-methylpropiophenone (Irgacure 2959), ascorbic acid, Triton X-100, HEPES, collagen I and III monoclonal antibodies (SAB4500362, SAB4500367), tenomodulin antibody (ABC305), basic fibroblast growth factor (bFGF), BMP-12, goat serum (GS), bovine serum albumin (BSA), and hexamethyldisilazane (HMDS) were obtained from Sigma-Aldrich. Alginate was kindly provided by FMC BioPolymer. hBM-MSCs isolated from the bone marrow of healthy adult donors were purchased from ATTC (Germany). Minimum essential medium alpha (α-MEM), fetal bovine serum (FBS), phosphate buffer solution (PBS), trypsin-EDTA, LIVE/DEAD Cell Viability Kit, l-glutamine, Alexa Fluor 488 Phalloidin (A12379), Alexa Fluor 488 antirabbit secondary antibody (A11034), and DRAQ5 (62254) were bought from Gibco Invitrogen (USA).

### Preparation of the Highly Aligned Hydrogel Fibers

For the synthesis of GelMA, 10% (w/v) porcine skin gelatin type A was dissolved in PBS at 60 °C and stirred at 240 rpm. Afterward, methacrylic anhydride was added drop by drop to the gelatin solution to attain a final concentration of 0.08% (v/v). The final solution was stirred for 3 h at 50 °C and then loaded in dialysis membranes (Spectro/Por molecular porous membrane tubing, MWCO 12–14000, Fisher Scientific). Dialysis was carried out for 10 days at 60 °C and 500 rpm. Lastly, the solution was freeze-dried, and lyophilized GelMA was obtained.

The bioink was prepared by dissolving 4% (w/v) of low molecular weight Alginate and 5% (w/v) lyophilized GelMA in HEPES containing 0.1% (w/v) Irgacure-2959 photoinitiator. The solution was then loaded with 20 million hBM-MSCs/mL and flown into a microfluidic coaxial needle extrusion system (inner needle: inner diameter (ID) = 0.25 mm, outer diameter (OD) = 0.50 mm; outer needle: ID 0.70 mm, OD = 1.00 mm). The flow rate of the cell-laden solution was set to 30 μL/min, while a calcium chloride solution was flown at 15 μL/min. As mentioned, during the wet-spinning, the hydrogel was in contact with calcium ions that allowed a first cross-linking of the fibers, which were then collected onto a drum with a rotation speed of 30 rpm. The fiber bundle was then subjected to a second cross-linking by exposure to 12.5 mW/cm^2^ UV light (Dymax BlueWave 75 UV Light Curing Spot Lamp, 365 nm, Torrington, CT).^36^

### Preparation of Electrospun Fibrous Matrix Loaded with BMP-12

For preparation of the electrospinning solution, 5% (w/v) MSPs were added to a mixed solvent system composed of 3:4 (v/v) acetic and formic acids. Subsequently, 12% (w/w) PCL and 17% (w/w) PA6 pellets were separately dissolved in the solution and mixed, obtaining a final ratio of 2:3 (w/w) of PCL and PA6, respectively.^35^ The electrospinning solution was loaded into 5 mL syringes and pumped through 22G blunted needles. The electrospinning flow rate was set at 0.20 mL/h, the working distance was fixed at 15 cm, and the applied voltage was 28 kV. After drying, the electrospun composite fibrous mats were loaded with BMP-12 by immersion in a solution of 0.01% (w/v) BMP-12 in PBS and shaken for 24 h.

### Preparation of the 3D Multilayered Structure

In order to obtain the 3D multilayered scaffolds, electrospun fibrous mats were coated by hydrogel yarns previously encapsulated with hBM-MSCs. The whole structure was then covered with 5% GelMA and cross-linked by 30 s exposition to 12.5 mW/cm^2^ UV light (Dymax BlueWave 75 UV Light Curing Spot Lamp, 365 nm, Torrington, CT).^20^

### Morphological Characterization

The morphology of the electrospun matrix, hydrogel fibers, 3D layered systems, and 3D layered system surgically sutured on tendon tissue was evaluated using SEM (Phenom, Holland). Images were acquired at 10 kV after sputtering the samples with gold. The diameter of the fibers was measured from microscope images using ImageJ (NIH, USA).

### Mechanical Properties

The mechanical properties of the electrospun matrix, hydrogel fibers, and electrospun matrix covered with hydrogel fibers were investigated by stretching the samples at a constant deformation rate of 10%/min by using the DMA Q800 instrument (TA Instruments, USA) equipped with tension clamps. All the specimens were preloaded to 0.001 N. Specimens’ Young’s moduli were identified in the initial quasi-linear range of the obtained stress–strain curves (up to 15% of strain).

### Release Assessment

The release of BMP-12 was evaluated for up to 30 days by incubating the composite electrospun matrix loaded with the growth factor in PBS. At each selected time point, the supernatant was collected and replaced with fresh PBS. The supernatant was then analyzed using an ELISA kit (LSBio) for BMP-12 detection to measure the protein concentration.

### Cell Studies

hBM-MSCs encapsulated into the hydrogel fibers, 3D layered systems, and 3D layered systems loaded with BMP-12 were cultured in alpha MEM supplemented with 10% FBS, 1% penicillin-streptomycin, 2 mM l-glutamine, 0.2 mM ascorbic acid, and 1 ng/mL bFGF for 14 days at 37 °C and 5% CO_2_.

#### Cell Viability

Cell viability was investigated by using a Live/Dead assay kit to evaluate the vitality of cells encapsulated within the scaffolds. After 24 h of culture, the structures were washed in HEPES, and 0.5 μL/mL calcein and 2 μL/mL ethidium homodimer were added. Calcein was introduced to stain viable cells in green, while ethidium homodimer was used to stain dead cells in red color. Scaffolds were incubated for 10 min at 37 °C and 5% CO_2_. Then, the constructs were washed in HEPES and imaged with a fluorescence microscope (Leica, USA). Alive and dead cells were detected from fluorescence images, and their respective number was calculated using the Cell Counter plugin of ImageJ (National Institutes of Health, USA).

#### Cell Morphology

Cell morphology was evaluated after 14 days of culture by staining actin filaments and cell nuclei using Alexa Fluor phalloidin and DRAQ5, respectively. Scaffolds were fixed using formalin 10% for 30 min and then washed thrice in HEPES for 5 min. Afterward, samples were treated with 0.3% (v/v) Triton X-100 in HEPES for 15 min and subsequently washed thrice. Then, 1% (w/v) BSA in HEPES was added for 30 min to inhibit the nonspecific binding, and incubation in 1:40 dilution of Alexa Fluor phalloidin in HEPES was performed for 40 min at room temperature (RT). Three washing steps were carried out. Subsequently, samples were treated with 1:1000 DRAQ5 solution in HEPES for 10 min and washed again. Imaging of the scaffolds was performed using a confocal microscope (Leica, USA). Cell orientation was quantified based on confocal images using ImageJ (National Institute of Health, USA) by measuring cell cytoskeletons orientation (ImageJ software, OrientationJ).

#### Immunohistochemistry

Collagen I and III expressions were investigated via immunohistochemistry to estimate the extracellular matrix (ECM) deposition. In contrast, tenomodulin expression was evaluated to assess the potential differentiation of hBM-MSCs toward tenogenic lines. Scaffolds were fixed after 14 days of culture in 10% formalin for 30 min. Afterward, 0.3% (v/v) Triton X-100 solution in HEPES was added for 15 min, and washing steps of 5 min were performed. Thirty min of incubation in 1% BSA-GS at room temperature was carried out to block the nonspecific staining. Then, the constructs were treated overnight at 4 °C with anticollagen I, anticollagen III, or antitenomodulin antibodies produced in rabbits (1:100 and 1:50 dilution for collagens and tenomodulin, respectively). Washings steps were performed. Subsequently, Alexa Fluor 488 antirabbit secondary antibody produced in goat solution (1:300 dilution) was added for 2 h at RT in the darkness. After washing, 10 min of incubation in DRAQ5 solution (1:1000) was carried out to stain the cell nuclei. The scaffolds were imaged with a confocal microscope (Leica, USA). ImageJ was used in order to run image quantification, measuring on the green channel the quantity of the expression area of collagen I, collagen III, or tenomodulin.

### *Ex Vivo* Testing

#### Sample Preparation and Mechanical Stretching

The 3D layered system was surgically sutured on a human Achilles tendon with two stitches. Achilles tendons were procured from 11 deceased male donors, aged 30–40, at the Department of Forensic Medicine, Medical University of Warsaw (Warsaw, Poland) up to 48 h after death. Donors were evaluated for tissue donation and procurement in accordance with Directive 2004/23/EC of the European Parliament and of the Council on setting standards of quality and safety for the donation, procurement, testing, processing, preservation, storage, and distribution of human tissues and cells and other related acts specifying the mode of operation of tissue and cell banks (OJ L 55, 9.4.2004) and Commission Directive 2006/17/EC of February 8, 2006 implementing Directive 2004/23/EC of the European Parliament and of the Council as regards certain technical requirements for the donation, procurement, and testing of human tissues and cells (OJ L 38, 9.2.2006). The procedure of processing Achilles tendon grafts was carried out in clean rooms (class C) of the tissue and cell bank. It included: mechanical cleaning of the collected tissues, rinsing and defatting the bone fragments of the calcaneal tubercle, and placing them in properly labeled double polyester-polyethylene packages, which were sealed with a thermal seal. Achilles tendons were radiation-sterilized by using an e-beam from the accelerator (LAE-10) with a beam power of 10.2 MeV at the Institute of Nuclear Chemistry and Technology in Warsaw (Poland) with a dose of 35 kGy.

The final constructs composed of the 3D layered system sutured on the tendon tissue were then mounted into a Ebers TC3 cyclic mechanical stretching machine (Ebers Medical; Spain). The cyclic test protocol provides stretching cycles between 2% and 4% strain (physiological range) for 5000 cycles at 1.4 Hz (frequency of normal walking).^49^

#### Microcomputer Tomography and SEM Analysis

Micro Computed Tomography (μCT) and SEM were used to visualize the integrity of the layered system when subjected to physiological mechanical stimulation. Prior to imaging, the samples were dehydrated using 50%, 70%, 90%, and 100% concentrated ethanol solutions (2 h each). Subsequently, samples were immersed in HMDS for 2 h and then dried in a fume hood overnight. For SEM analysis, samples were coated with a thin layer of gold and imaged at 10 kV. For μCT scanning, samples subjected to cyclic mechanical stretching were compared to nonstretched samples, and analyzed in the area between the two surgical stitches by using Xradia MicroXCT-400. The scanning parameters were set as 40 kV voltage, 10 W power, no filter material, 0.18° rotation step in an angle interval of 184°.^50^ The voxel size was 5.2 × 5.2 × 5.2 μm^3^. Image analysis and 3D reconstruction of the samples were implemented with Avizo 3D software (Thermo Fisher Scientific).

### Statistical Analysis

Samples were analyzed at least in triplicate unless specified, and data were expressed as mean ± standard deviation. Experimental data were statistically analyzed through one-way ANOVA analysis followed by a Tukey’s multiple pairwise comparisons test calculated by Origin 8. Values are reported as statistically significant when *p* ≤ 0.05: *p ≤ 0.05, **p ≤ 0.01, ***p ≤ 0.001, and ****p ≤ 0.0001.

## Supporting Information Available

Schematic illustration of mechanical testing; release of BMP-12 from pristine polymeric matrix; cell viability of mesenchymal stem cells encapsulated within the constructs after 24 h of culture; μCT images of constructs surgically sutured on tendon tissue: not mechanically stretched *vs* cyclic mechanically stretched (PDF)

## References

[ref1] Lomas A. J.; Ryan C. N. M.; Sorushanova A.; Shologu N.; Sideri A. I.; Tsioli V.; Fthenakis G. C.; Tzora A.; Skoufos I.; Quinlan L. R.; O’Laighin G.; Mullen A. M.; Kelly J. L.; Kearns S.; Biggs M.; Pandit A.; Zeugolis D. I. The Past, Present and Future in Scaffold-Based Tendon Treatments. Adv. Drug Delivery Rev. 2015, 84, 257–277. 10.1016/j.addr.2014.11.022. 25499820

[ref2] Walden G.; Liao X.; Donell S.; Raxworthy M. J.; Riley G.; Saeed A. A Clinical, Biological and Biomaterials Perspective into Tendon Injuries and Regeneration. Tissue Eng. Part B Rev. 2017, 23 (1), 44 10.1089/ten.teb.2016.0181. 27596929 PMC5312458

[ref3] Langer R.; Vacanti J. P. Tissue Engineering. Science (80-.). 1993, 260 (May), 920–926. 10.1126/science.8493529. 8493529

[ref4] Costantini M.; Testa S.; Rinoldi C.; Celikkin N.; Idaszek J.; Colosi C.; Barbetta A.; Gargioli C.; Święszkowski W. 3D Tissue Modelling of Skeletal Muscle Tissue. In Biofabrication and 3D Tissue Modeling; The Royal Society of Chemistry, 2019; Chapter 9, pp 184–215. 10.1039/9781788012683-00184.

[ref5] Sardelli L.; Pacheco D. P.; Zorzetto L.; Rinoldi C.; Święszkowski W.; Petrini P. Engineering Biological Gradients. J. Appl. Biomater. Funct. Mater. 2019, 17 (1), 1–15. 10.1177/2280800019829023. 30803308

[ref6] Tamayol A.; Akbari M.; Annabi N.; Paul A.; Khademhosseini A.; Juncker D. Fiber-Based Tissue Engineering: Progress, Challenges, and Opportunities. Biotechnol. Adv. 2013, 31 (5), 669–687. 10.1016/j.biotechadv.2012.11.007. 23195284 PMC3631569

[ref7] Wang Y.; Jin S.; Luo D.; He D.; Shi C.; Zhu L.; Guan B.; Li Z.; Zhang T.; Zhou Y.; Wang C.; Liu Y. Functional Regeneration and Repair of Tendons Using Biomimetic Scaffolds Loaded with Recombinant Periostin. Nat. Commun. 2021, 12, 1–19. 10.1038/s41467-021-21545-1. 33637721 PMC7910464

[ref8] Kijeńska E.; Swieszkowski W. 2 - General Requirements of Electrospun Materials for Tissue Engineering: Setups and Strategy for Successful Electrospinning in Laboratory and Industry. In Electrospun Materials for Tissue Engineering and Biomedical Applications; Uyar T., Kny E., Eds.; Woodhead Publishing, 2017; pp 43–56. 10.1016/B978-0-08-101022-8.00002-8.

[ref9] Lagaron J. M.; Solouk A.; Castro S.; Echegoyen Y. 3 - Biomedical Applications of Electrospinning, Innovations, and Products; In Electrospun Materials for Tissue Engineering and Biomedical Applications; Uyar T., Kny E., Eds.; Woodhead Publishing, 2017; pp 57–72. 10.1016/B978-0-08-101022-8.00010-7.

[ref10] Liu H.; Chansoria P.; Delrot P.; Angelidakis E.; Rizzo R.; Rütsche D.; Applegate L. A.; Loterie D.; Zenobi-Wong M. Filamented Light (FLight) Biofabrication of Highly Aligned Tissue-Engineered Constructs. Adv. Mater. 2022, 34 (45), 2204301 10.1002/adma.202204301. 36095325

[ref11] Liu S.; Qin M.; Hu C.; Wu F.; Cui W.; Jin T.; Fan C. Tendon Healing and Anti-Adhesion Properties of Electrospun Fibrous Membranes Containing BFGF Loaded Nanoparticles. Biomaterials 2013, 34 (19), 4690–4701. 10.1016/j.biomaterials.2013.03.026. 23541108

[ref12] Beason D. P.; Connizzo B. K.; Dourte L. M.; Mauck R. L.; Soslowsky L. J.; Steinberg D. R.; Bernstein J. Fiber-Aligned Polymer Scaffolds for Rotator Cuff Repair in a Rat Model. J. Shoulder Elb. Surg. 2012, 21 (2), 245–250. 10.1016/j.jse.2011.10.021. 22244068

[ref13] Yin Z.; Sun L.; Shi L.; Nie H.; Dai J.; Zhang C. Bioinspired Bimodal Micro-Nanofibrous Scaffolds Promote the Tenogenic Differentiation of Tendon Stem/Progenitor Cells for Achilles Tendon Regeneration. Biomater. Sci. 2022, 10 (3), 753–769. 10.1039/D1BM01287H. 34985056

[ref14] Yang G.; Lin H.; Rothrauff B. B.; Yu S.; Tuan R. S. Multilayered Polycaprolactone/Gelatin Fiber-Hydrogel Composite for Tendon Tissue Engineering. Acta Biomater. 2016, 35, 68–76. 10.1016/j.actbio.2016.03.004. 26945631 PMC5408748

[ref15] Xie Y.; Zhang F.; Akkus O.; King M. W. A Collagen/PLA Hybrid Scaffold Supports Tendon-Derived Cell Growth for Tendon Repair and Regeneration. J. Biomed. Mater. Res. Part B Appl. Biomater. 2022, 110 (12), 2624–2635. 10.1002/jbm.b.35116. PMC9795886 35779243

[ref16] Costa-Almeida R.; Domingues R. M. A.; Fallahi A.; Avci H.; Yazdi I. K.; Akbari M.; Reis R. L.; Tamayol A.; Gomes M. E.; Khademhosseini A. Cell-Laden Composite Suture Threads for Repairing Damaged Tendons. J. Tissue Eng. Regen. Med. 2018, 12 (4), 1039–1048. 10.1002/term.2605. 29115019 PMC6594050

[ref17] Ma H.; Yang C.; Ma Z.; Wei X.; Younis M. R.; Wang H.; Li W.; Wang Z.; Wang W.; Luo Y.; Huang P.; Wang J. Multiscale Hierarchical Architecture-Based Bioactive Scaffolds for Versatile Tissue Engineering. Adv. Healthc. Mater. 2022, 11 (13), 2102837 10.1002/adhm.202102837. 35355444

[ref18] Pardo A.; Bakht S. M.; Gomez-Florit M.; Rial R.; Monteiro R. F.; Teixeira S. P. B.; Taboada P.; Reis R. L.; Domingues R. M. A.; Gomes M. E. Magnetically-Assisted 3D Bioprinting of Anisotropic Tissue-Mimetic Constructs. Adv. Funct. Mater. 2022, 32, 2208940 10.1002/adfm.202208940.

[ref19] Chainani A.; Hippensteel K. J.; Kishan A.; Garrigues N. W.; Ruch D. S.; Guilak F.; Little D. Multilayered Electrospun Scaffolds for Tendon Tissue Engineering. Tissue Eng. Part A 2013, 19 (23–24), 2594–2604. 10.1089/ten.tea.2013.0165. 23808760 PMC3856877

[ref20] Rinoldi C.; Fallahi A.; Yazdi I.; Campos Paras J.; Kijeńska-Gawrońska E.; Trujillo-de Santiago G.; Tuoheti A.; Demarchi D.; Annabi N.; Khademhosseini A.; Swieszkowski W.; Tamayol A. Mechanical and Biochemical Stimulation of 3D Multi-Layered Scaffolds for Tendon Tissue Engineering. ACS Biomater. Sci. Eng. 2019, 5 (6), 2953–2964. 10.1021/acsbiomaterials.8b01647. 33405598

[ref21] Chae S.; Choi Y.-J.; Cho D.-W. Mechanically and Biologically Promoted Cell-Laden Constructs Generated Using Tissue-Specific Bioinks for Tendon/Ligament Tissue Engineering Applications. Biofabrication 2022, 14 (2), 025013 10.1088/1758-5090/ac4fb6. 35086074

[ref22] Yao Z.; Qian Y.; Jin Y.; Wang S.; Li J.; Yuan W.-E.; Fan C. Biomimetic Multilayer Polycaprolactone/Sodium Alginate Hydrogel Scaffolds Loaded with Melatonin Facilitate Tendon Regeneration. Carbohydr. Polym. 2022, 277, 118865 10.1016/j.carbpol.2021.118865. 34893270

[ref23] Rinoldi C.; Kijeńska-Gawrońska E.; Khademhosseini A.; Tamayol A.; Swieszkowski W. Fibrous Systems as Potential Solutions for Tendon and Ligament Repair, Healing and Regeneration. Adv. Healthc. Mater. 2021, 10, 2001305 10.1002/adhm.202001305. PMC8048718 33576158

[ref24] Calejo I.; Labrador-Rached C. J.; Gomez-Florit M.; Docheva D.; Reis R. L.; Domingues R. M. A.; Gomes M. E. Bioengineered 3D Living Fibers as In Vitro Human Tissue Models of Tendon Physiology and Pathology. Adv. Healthc. Mater. 2022, 11 (15), 2102863 10.1002/adhm.202102863. 35596614

[ref25] Ning C.; Gao C.; Li P.; Fu L.; Chen W.; Liao Z.; Xu Z.; Yuan Z.; Guo W.; Sui X.; Liu S.; Guo Q. Dual-Phase Aligned Composite Scaffolds Loaded with Tendon-Derived Stem Cells for Achilles Tendon Repair. Adv. Ther. 2022, 5 (9), 2200081 10.1002/adtp.202200081.

[ref26] Donderwinkel I.; Tuan R. S.; Cameron N. R.; Frith J. E. Tendon Tissue Engineering: Current Progress towards an Optimized Tenogenic Differentiation Protocol for Human Stem Cells. Acta Biomater. 2022, 145, 25–42. 10.1016/j.actbio.2022.04.028. 35470075

[ref27] Xue Y.; Kim H.-J.; Lee J.; Liu Y.; Hoffman T.; Chen Y.; Zhou X.; Sun W.; Zhang S.; Cho H.-J.; Lee J.; Kang H.; Ryu W.; Lee C.-M.; Ahadian S.; Dokmeci M. R.; Lei B.; Lee K.; Khademhosseini A. Co-Electrospun Silk Fibroin and Gelatin Methacryloyl Sheet Seeded with Mesenchymal Stem Cells for Tendon Regeneration. Small 2022, 18 (21), 2107714 10.1002/smll.202107714. PMC9714686 35487761

[ref28] Thomopoulos S.; Das R.; Sakiyama-Elbert S.; Silva M. J.; Charlton N.; Gelberman R. H. BFGF and PDGF-BB for Tendon Repair: Controlled Release and Biologic Activity by Tendon Fibroblasts in Vitro. Ann. Biomed. Eng. 2010, 38 (2), 225–234. 10.1007/s10439-009-9844-5. 19937274 PMC2843401

[ref29] Gonçalves A. I.; Rodrigues M. T.; Lee S.-J.; Atala A.; Yoo J. J.; Reis R. L.; Gomes M. E. Understanding the Role of Growth Factors in Modulating Stem Cell Tenogenesis. PLoS One 2013, 8 (12), e83734 10.1371/journal.pone.0083734. 24386267 PMC3875481

[ref30] Zarychta-Wiśniewska W.; Burdzinska A.; Kulesza A.; Gala K.; Kaleta B.; Zielniok K.; Siennicka K.; Sabat M.; Paczek L. Bmp-12 Activates Tenogenic Pathway in Human Adipose Stem Cells and Affects Their Immunomodulatory and Secretory Properties. BMC Cell Biol. 2017, 18 (1), 1–14. 10.1186/s12860-017-0129-9. 28214472 PMC5316159

[ref31] Jelinsky S. A.; Li L.; Ellis D.; Archambault J.; Li J.; St. Andre M.; Morris C.; Seeherman H. Treatment with RhBMP12 or RhBMP13 Increase the Rate and the Quality of Rat Achilles Tendon Repair. J. Orthop. Res. 2011, 29 (10), 1604–1612. 10.1002/jor.21427. 21469182

[ref32] Lee J. Y.; Zhou Z.; Taub P. J.; Ramcharan M.; Li Y.; Akinbiyi T.; Maharam E. R.; Leong D. J.; Laudier D. M.; Ruike T.; Torina P. J.; Zaidi M.; Majeska R. J.; Schaffler M. B.; Flatow E. L.; Sun H. B. BMP-12 Treatment of Adult Mesenchymal Stem Cells In Vitro Augments Tendon-like Tissue Formation and Defect Repair In Vivo. PLoS One 2011, 6 (3), e17531 10.1371/journal.pone.0017531. 21412429 PMC3055887

[ref33] Fu S. C.; Wong Y. P.; Chan B. P.; Pau H. M.; Cheuk Y. C.; Lee K. M.; Chan K.-M. The Roles of Bone Morphogenetic Protein (BMP) 12 in Stimulating the Proliferation and Matrix Production of Human Patellar Tendon Fibroblasts. Life Sci. 2003, 72 (26), 2965–2974. 10.1016/S0024-3205(03)00169-3. 12706484

[ref34] Cheng X.; Tsao C.; Sylvia V. L.; Cornet D.; Nicolella D. P.; Bredbenner T. L.; Christy R. J. Platelet-Derived Growth-Factor-Releasing Aligned Collagen-Nanoparticle Fibers Promote the Proliferation and Tenogenic Differentiation of Adipose-Derived Stem Cells. Acta Biomater. 2014, 10 (3), 1360–1369. 10.1016/j.actbio.2013.11.017. 24291329

[ref35] Rinoldi C.; Kijeńska E.; Chlanda A.; Choinska E.; Khenoussi N.; Tamayol A.; Khademhosseini A.; Swieszkowski W. Nanobead-on-String Composites for Tendon Tissue Engineering. J. Mater. Chem. B 2018, 6 (19), 3116–3127. 10.1039/C8TB00246K. 32254346

[ref36] Rinoldi C.; Costantini M.; Kijeńska E.; Heljak M.; Monika C.; Buda R.; Baldi J.; Cannata S.; Guzowski J.; Gargioli C.; Khademhosseini A.; Swieszkowski W. Tendon Tissue Engineering : Effects of Mechanical and Biochemical Stimulation on Stem Cell Alignment on Cell-Laden Hydrogel Yarns. Adv. Healthc. Mater. 2019, 8, 1801218 10.1002/adhm.201801218. 30725521

[ref37] Itoi E.; Berglund L. J.; Grabowski J. J.; Schultz F. M.; Growney E. S.; Morrey B. F.; An K.-N. Tensile Properties of the Supraspinatus Tendon. J. Orthop. Res. 1995, 13 (4), 578–584. 10.1002/jor.1100130413. 7674074

[ref38] Violini S.; Ramelli P.; Pisani L. F.; Gorni C.; Mariani P. Horse Bone Marrow Mesenchymal Stem Cells Express Embryo Stem Cell Markers and Show the Ability for Tenogenic Differentiation by in Vitro Exposure to BMP-12. BMC Cell Biol. 2009, 10, 29 10.1186/1471-2121-10-29. 19383177 PMC2678092

[ref39] Li Z. Y.; Liu Y.; Wang X. Q.; Liu L. H.; Hu J. J.; Luo G. F.; Chen W. H.; Rong L.; Zhang X. Z. One-Pot Construction of Functional Mesoporous Silica Nanoparticles for the Tumor-Acidity-Activated Synergistic Chemotherapy of Glioblastoma. ACS Appl. Mater. Interfaces 2013, 5 (16), 7995–8001. 10.1021/am402082d. 23869943

[ref40] Tao X.; Yang Y. J.; Liu S.; Zheng Y. Z.; Fu J.; Chen J. F. Poly(Amidoamine) Dendrimer-Grafted Porous Hollow Silica Nanoparticles for Enhanced Intracellular Photodynamic Therapy. Acta Biomater. 2013, 9 (5), 6431–6438. 10.1016/j.actbio.2013.01.028. 23380206

[ref41] Kortesuo P.; Ahola M.; Kangas M.; Leino T.; Laakso S.; Vuorilehto L.; Yli-Urpo A.; Kiesvaara J.; Marvola M. Alkyl-Substituted Silica Gel as a Carrier in the Controlled Release of Dexmedetomidine. J. Controlled Release 2001, 76 (3), 227–238. 10.1016/S0168-3659(01)00428-X. 11578738

[ref42] Datt A.; El-Maazawi I.; Larsen S. C. Aspirin Loading and Release from MCM-41 Functionalized with Aminopropyl Groups via Co-Condensation or Postsynthesis Modification Methods. J. Phys. Chem. C 2012, 116 (34), 18358–18366. 10.1021/jp3063959.

[ref43] Zhang Y.; Zhi Z.; Jiang T.; Zhang J.; Wang Z.; Wang S. Spherical Mesoporous Silica Nanoparticles for Loading and Release of the Poorly Water-Soluble Drug Telmisartan. J. Controlled Release 2010, 145 (3), 257–263. 10.1016/j.jconrel.2010.04.029. 20450945

[ref44] Wang W.; Chen S.; Zhang L.; Wu X.; Wang J.; Chen J. F.; Le Y. Poly(Lactic Acid)/Chitosan Hybrid Nanoparticles for Controlled Release of Anticancer Drug. Mater. Sci. Eng., C 2015, 46, 514–520. 10.1016/j.msec.2014.10.048. 25492016

[ref45] Laha A.; Sharma C. S.; Majumdar S. Sustained Drug Release from Multi-Layered Sequentially Crosslinked Electrospun Gelatin Nanofiber Mesh. Mater. Sci. Eng., C 2017, 76, 782–786. 10.1016/j.msec.2017.03.110. 28482590

[ref46] Zhang P.; Zardán Gómez De La Torre T.; Forsgren J.; Bergström C. A. S.; Strømme M. Diffusion-Controlled Drug Release from the Mesoporous Magnesium Carbonate Upsalite®. J. Pharm. Sci. 2016, 105 (2), 657–663. 10.1002/jps.24553. 26087956

[ref47] Stewart C. A.; Finer Y.; Hatton B. D. Drug Self-Assembly for Synthesis of Highly-Loaded Antimicrobial Drug-Silica Particles. Sci. Rep. 2018, 8 (1), 1–12. 10.1038/s41598-018-19166-8. 29343729 PMC5772632

[ref48] Hoffmann A.; Gross G. Tendon and Ligament Engineering: From Cell Biology to in Vivo Application. Regen. Med. 2006, 1 (4), 563–574. 10.2217/17460751.1.4.563. 17465850

[ref49] Chandrashekar N.; Slauterbeck J.; Hashemi J. Effects of Cyclic Loading on the Tensile Properties of Human Patellar Tendon. Knee 2012, 19 (1), 65–68. 10.1016/j.knee.2010.11.014. 21216601

[ref50] Costantini M.; Testa S.; Mozetic P.; Barbetta A.; Fuoco C.; Fornetti E.; Tamiro F.; Bernardini S.; Jaroszewicz J.; Święszkowski W.; Trombetta M.; Castagnoli L.; Seliktar D.; Garstecki P.; Cesareni G.; Cannata S.; Rainer A.; Gargioli C. Microfluidic-Enhanced 3D Bioprinting of Aligned Myoblast-Laden Hydrogels Leads to Functionally Organized Myofibers in Vitro and in Vivo. Biomaterials 2017, 131, 98–110. 10.1016/j.biomaterials.2017.03.026. 28388499

