# Supplementary material for: Mesoporous Particle Embedded Nanofibrous Scaffolds Sustain Biological Factors for Tendon Tissue Engineering
Source: ACS Mater Au. 2023 Jul 24;3(6):636–45. doi: 10.1021/acsmaterialsau.3c00012 (PMC10636765; doi:10.1021/acsmaterialsau.3c00012)
Supplement: Supplementary file 1 — mg3c00012_si_001.pdf [file mg3c00012_si_001.pdf]

Supporting information

Mesoporous particle embedded nanofibrous scaffolds sustain biological factors for tendon tissue engineering

Chiara Rinoldi[a,b], Ewa Kijeńska-Gawrońska[a,c], Marcin Heljak[a], Jakub Jaroszewicz[a], Artur Kamiński[d], Ali Khademhosseini[e,f,g], Ali Tamayol[h,i], Wojciech Swieszkowski[a]*

[a]Faculty of Materials Science and Engineering, Warsaw University of Technology, Warsaw, 02-507, Poland.

[b]Institute of Fundamental Technological Research, Polish Academy of Sciences, Warsaw, 02-106, Poland.

[c]Centre for Advanced Materials and Technologies CEZAMAT, Warsaw University of Technology, Warsaw, 02-822, Poland.

[d]Department of Transplantology and Central Tissue Bank, Medical University of Warsaw, Warsaw, 02-091, Poland.

[e]Department of Bioengineering, University of California, Los Angeles, CA, 90095, United States.

[f]California NanoSystems Institute, University of California, Los Angeles, CA, 90095, United States.

[g]Terasaki Institute for Biomedical Innovation, Los Angeles, CA, 90024, United States.

[h]Department of Mechanical and Materials Engineering, University of Nebraska, Lincoln, NE, 68588, United States.

[i]Department of Biomedical Engineering, University of Connecticut Health Center, Farmington, CT, 06030, United States.

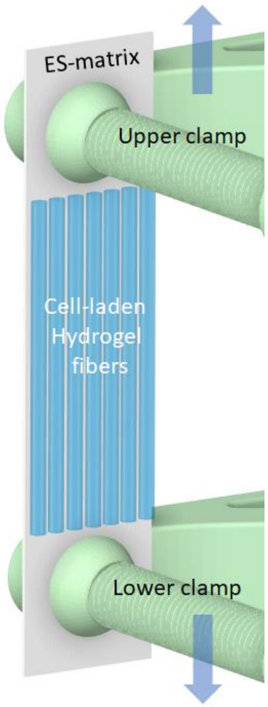

Figure S1. Schematic illustration of specimens' fixation during mechanical testing of electrospun matrix covered with hydrogel fibers.

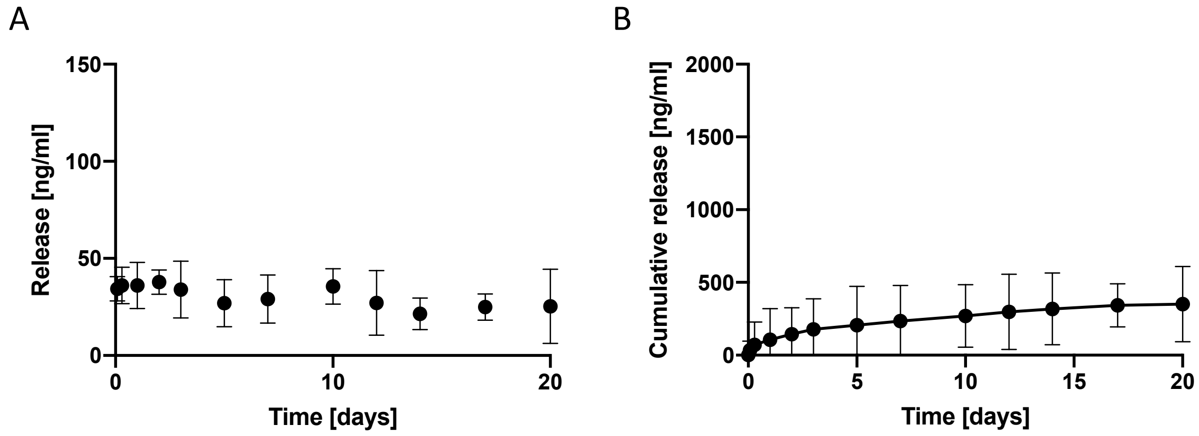

Figure S2. Release of BMP-12 from the pristine polymeric matrix with no mesoporous silica embedded: daily release (A) and cumulative release (B).

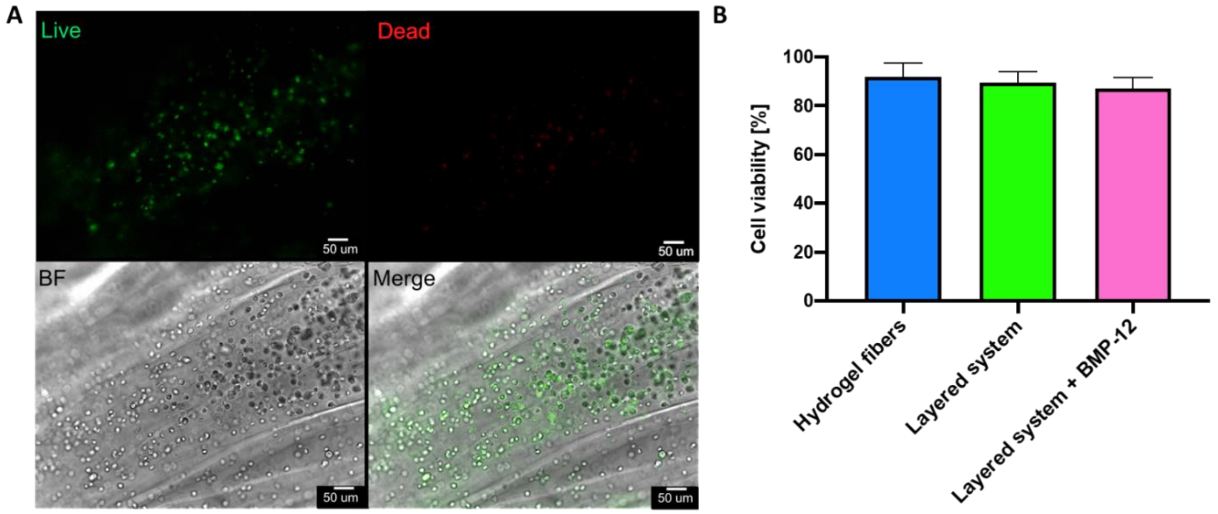

Figure S3. Cell viability of mesenchymal stem cells encapsulated within the hydrogel fibers, 3D layered system, and 3D layered system loaded with BMP-12 after 24 hours of culture. A) Representative image of alive (green) and dead (red) hBM-MSCs loaded into the layered system loaded with BMP-12; B) quantification of cell viability in the scaffolds.

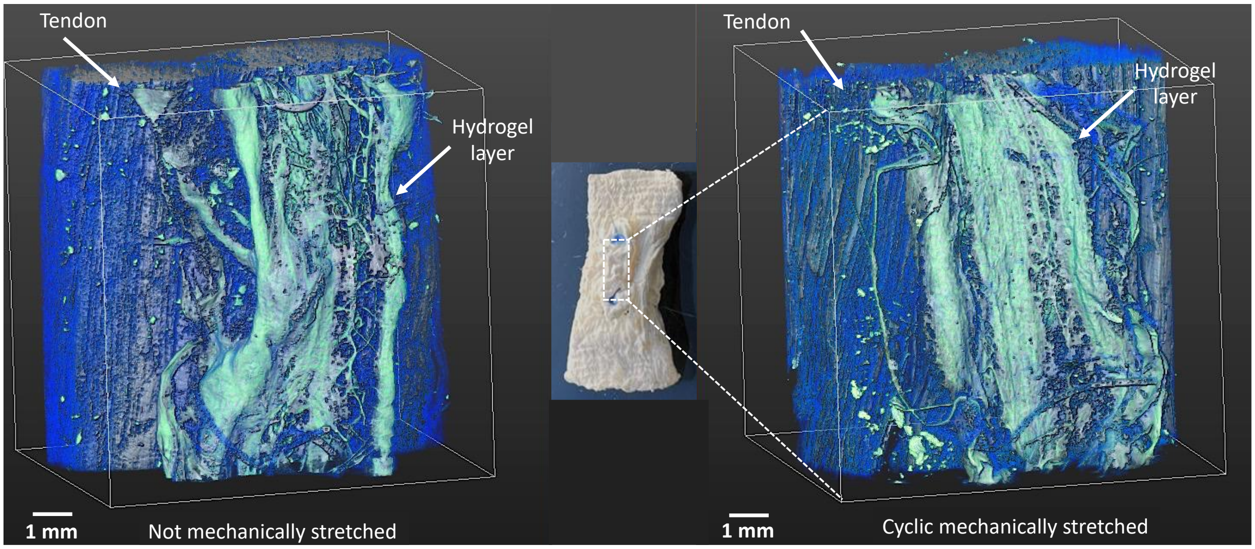

Figure S4. μCT images of constructs surgically sutured on tendon tissue: not mechanically stretched (left) *vs.* cyclic mechanically stretched (right). The area of imaging was individuated between the two surgical stitches (as defined in the macroscopic picture in the center). Tendon and hydrogel layers are pointed by the arrows, showing the maintained integrity of the hydrogel layer when subjected to mechanical loading.
